# How to Develop and Deliver a Virtual Exchange Based on Collaborative Online International Learning Principles (COIL)

**DOI:** 10.1111/tct.70121

**Published:** 2025-06-19

**Authors:** Jonathan Kennedy, Jessie Dubreuil, Danielle Thibodeau, Aaron Zachmeier

**Affiliations:** ^1^ Queen Mary University of London London UK; ^2^ UC Santa Cruz Santa Cruz California USA; ^3^ The Nueva School California USA; ^4^ Kuehne Logistics University and University of East Anglia Hamburg Germany

**Keywords:** collaborative online international learning, intercultural competence, virtual international exchanges

## Abstract

Collaborative Online International Learning (COIL) is a pedagogical approach to virtual exchanges that emphasises experiential and collaborative learning activities. The key aim of COIL virtual exchanges is to encourage students to develop intercultural competence—that is, the ability to communicate and cooperate with people from different backgrounds. Intercultural competence is a particularly important skill for doctors and other health workers, who will spend much of their working lives interacting with patients and colleagues who have been shaped by different values, beliefs and experiences. Many nonmedical education studies demonstrate that COIL virtual exchanges have a positive impact on students' intercultural competence. Recently, a couple of studies have shown that this is also true for medical students. Despite this, COIL virtual exchanges are not widely utilised in medical education. This paper outlines some of the key steps clinical teachers must take when designing and delivering a virtual exchange based on COIL principles. It is grounded in the literature on COIL virtual exchanges, as well as our experience of designing, delivering and evaluating a COIL virtual exchange involving medical students in the United Kingdom and pre‐med students in the United States. The paper provides practical guidance covering: finding an exchange partner; choosing a focus for your exchange; and designing an exchange that creates a space for students to work together and foster creativity, critical thinking and problem‐solving abilities.

## 1 Introduction

Virtual exchanges use online tools and platforms to allow students located in different countries to learn together. Since these collaborations do not involve travel, they are much more accessible than traditional in‐person student exchanges—that is, study abroad programmes. Virtual exchanges can be particularly beneficial for medical students because the intensity and inflexibility of their courses are not conducive to spending a semester or year overseas.

BOX 1 **What is Collaborative Online International Learning (COIL)?**

1. **Virtual exchanges** use online tools and platforms to allow students located in different countries to learn together.
2. **COIL** is a pedagogical approach to virtual exchanges that emphasises the importance of **experiential and collaborative learning activities**.
3. Faculty **collaborate on designing** the virtual exchange, and students engage in **collaborative projects** that encourage problem‐solving, critical thinking and creativity.
4. The main aim of COIL virtual exchanges is to develop students' **intercultural competence**, the ability to communicate and cooperate with people from different backgrounds.
5. COIL also helps students to better **understand** their own and other societies and to develop skills like **teamwork and digital literacy.**

*Virtual exchanges can be particularly beneficial for medical students*.

**FIGURE 1:**
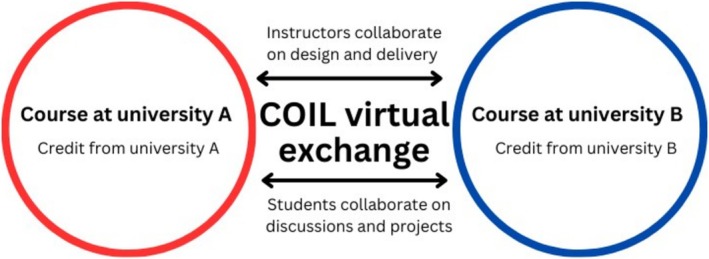
A graphical representation of a COIL virtual exchange.

Collaborative Online International Learning (COIL) is a specific pedagogical approach to virtual exchanges. Faculty from two or more cultures work together to develop an exchange programme. Students still follow the curricula at their home universities, and the virtual exchange is designed to complement their existing knowledge‐ and skill‐base. COIL virtual exchanges are usually a compulsory component of the curriculum that is designed to consolidate and apply what students learn in the classroom. They can range in length from a few weeks to a semester or longer. Their key characteristic is the emphasis on experiential and collaborative learning activities that encourage students to communicate and cooperate with people from different backgrounds in order to improve digital literacy, exchange knowledge, and, above all, develop intercultural competence [1]. The basic features of a COIL virtual exchange are depicted in graphical form in Figure 1.

COIL virtual exchanges are an increasingly popular pedagogical tool in higher education but are not widely utilised in clinical education. PubMed returns just three results for COIL virtual exchanges involving medical students; and all three articles focus on the same exchange between pre‐med students in the United States and medical students in Egypt [2, 3, 4]. Nevertheless, medical students have a great deal to gain from taking part in COIL virtual exchanges.

*COIL virtual exchanges are an increasingly popular pedagogical tool in higher education but are not widely utilised in clinical education*.

Medical students must master an enormous body of factual material and a wide range of technical skills to be able to provide patients with effective and safe care [5]. In recent years, there have been important innovations in clinical education, including the move towards more engaged learning through project‐based and team‐based approaches. Despite this, many clinical training programmes still bear an uncanny resemblance to what Freire calls the ‘banking model of education,’ in which learning is conceptualised as the act of teachers depositing knowledge in students' heads, ‘which the students patiently receive, memorize and repeat’ [6].

Less tangible but nevertheless crucial skills can be overlooked by clinical teachers because of the necessary focus on teaching facts and techniques. Intercultural competence is a particularly important skill for doctors and other health workers, as clinicians spend much of their working lives interacting with patients and colleagues who have been shaped by different values, beliefs and experiences. There is some evidence that intercultural competence improves healthcare quality and outcomes [7].

This paper outlines some of the key issues that clinical teachers must consider when designing and delivering a virtual exchange based on COIL principles. It is informed by pedagogical literature, as well as our own experience in designing and delivering an exchange between medical students based in east London, UK, and undergraduate students on an interdisciplinary global and community health programme in northern California. This exchange was centred on a 5‐week‐long simulated pandemic exercise.

## 2 Getting Started

Finding one or more partner institutions is the crucial first step in developing a virtual exchange. Professional networks are one source of potential collaborators. Your university's international office may be able to help identify potential partners. Bodies like Erasmus+ and the Fulbright Commission run programmes that support virtual exchanges. Our partnership was initially supported by the US‐UK Fulbright Commission's Global Challenges Teaching Award programme.

The partners' locations will have a profound influence on the character of the intercultural exchange. A UK–US collaboration worked well for our simulated pandemic exercise. We designed activities that, inter alia, compared: the unequal impact of infectious disease on particular socio‐economic groups; the differences between British and American health systems; and popular attitudes to public health responses, like lockdowns and vaccine and mask mandates. Despite the similarities between the two cultures, the differences in experiences and expectations were significant enough to allow students to learn a great deal from these collaborative activities.

In some instances, it will be better for the partners to be located in very different socio‐economic contexts. After all, medical students from high‐income countries need to understand the issues associated with the provision of healthcare in low‐income settings; and such exchanges also benefit students from the ‘majority world’ who have limited opportunities to improve their intercultural competence through in‐person exchanges because of financial and visa‐related barriers to travel [8].

We suggest that clinical teachers extend the concept of intercultural competence to encompass the ability to interact with students and faculty from nonclinical backgrounds—that is, other academic cultures. Research shows that medical students ‘benefit from interdisciplinary education with outcome effects primarily relating to changes in knowledge, skills, attitudes, and beliefs’ [9]. Although there will be instances when it is best to partner with another medical school, there are also benefits to choosing partners based in another discipline.

## 3 Design and Delivering a COIL Virtual Exchange

Another fundamental step in designing a COIL virtual exchange is deciding on the focus of the collaboration. It is best to choose subject areas that involve so‐called ‘wicked problems’—that is, complex, multifaceted issues characterised by ambiguity, interconnectedness and the absence of a clear and definitive solution [10]. Examples of wicked problems include climate change, poverty, inequality and pandemics.

Wicked problems provide opportunities for students to discuss their diverse perspectives and work together on policy‐oriented collaborative activities. Parts of the medical school curricula that focus on wicked problems include public health, global health and planetary health [11]. To engage with these topics in a meaningful way, students must think beyond biomedical causes and solutions to illness and disease, and instead explore the broader determinants of health.

BOX 2 **Ideas for making COIL virtual exchanges relevant to medical students.**

1. Focus on ‘**wicked problems’** in order encourage students to look beyond biomedical perspectives and instead use an **interdisciplinary lens** that considers the **upstream determinants of health**.
2. Design experiential activities to **consolidate students' learning** in their respective programmes and facilitate the **exchange of country‐specific knowledge and experiences**.
3. Ensure that the activities create a space for students to work together to **foster creativity, critical thinking and problem‐solving abilities**.
4. Extend the **concept of intercultural competence** to include the ability to communicate and cooperate with students and faculty from **nonclinical backgrounds**.
5. Challenge learners to present their ideas in a variety of **nontraditional formats** that develop **digital literacy** and other competencies.
6. Clearly **articulate the aims and benefits of the COIL approach** throughout the course of the virtual exchange, and focus on their applicability to medical students' future career.

COIL emphasises an experiential approach that allows students to apply knowledge to real‐world situations. It aims to deepen understanding of a topic, as well as foster creativity, critical thinking and problem‐solving abilities [12]. With this in mind, we designed a five‐week virtual exchange based on a simulated pandemic response exercise.

*COIL emphasises an experiential approach that allows students to apply knowledge to real‐world situations*.

The classes were split into groups of around five or six that included students from both universities. Each group played the role of a World Health Organisation (WHO) Strategic Advisory Group of Experts (SAGE). Every week the students were sent a video featuring the (fictional) WHO's Assistant Director‐General for Pandemic Response, who provided an update on the progress of the pandemic and set a collaborative activity with associated deliverables. The tasks were designed to achieve COIL objectives outlined above: consolidate the students' learning in their respective programmes; facilitate the exchange of country‐specific knowledge and experiences; and create a space for students to work together and develop their intercultural competence.

BOX 3 **An example timetable for a COIL virtual exchange on the theme of pandemics.**

*Week 1. Introduction and ‘icebreaker’*. Students record and share a 3‐min ‘health narrative’ video in which they reflect on their personal experience of illness and disease. The concept of COIL virtual exchanges and the simulated pandemic are introduced, and students are assigned to SAGEs (Strategic Advisory Group of Experts).

*Week 2. The social determinants of health*. Each SAGE collaborates to create photo essays that illustrate social and economic inequalities in their local areas and use the literature on social determinants of health to explore how these might impact the progress of a pandemic.

*Week 3. Public health interventions (i)*. Using personal experiences from COVID‐19 and evidence from different country case studies, SAGE members interview each other on the pros and cons of lockdowns as a public health strategy—paying notice to the WHO's definition of health as ‘a state of complete physical, mental and social well‐being and not merely the absence of disease or infirmity’.

*Week 4. Public health interventions (ii)*. Each SAGE creates a poster that aims to increase vaccine uptake among a specified marginalised group. The students present their posters to the other SAGEs, justifying their design by referring to topics discussed in weeks 2 and 3, as well as the public health literature.

*Week 5. Reflection*. Each SAGE reflects on the virtual exchange in a PechaKucha—a presentation format comprising 20 slides, each with 20 s of commentary. Students were asked to focus on three questions: What have you learned from collaborating with students from another culture? What challenges did you face? How will you apply what you have learned from this experience in your future studies, career or personal life?

The exercises were designed to allow students to develop technical skills that may be beneficial in their careers, but which are not covered elsewhere in the curriculum. The tasks included getting students to: explore their own and their peers' experience of illness and diseases by recording and sharing a ‘health narrative’ video, then watching and commenting on other videos; create photo essays to illustrate social inequalities in their respective locales with commentary on how this might impact the progress of the pandemic; interview each other on the pros and cons of lockdowns as a public health strategy; design posters to increase vaccine uptake in marginalised groups; and perform a PechaKucha—a presentation format comprising 20 slides, each with 20 s of commentary—that reflected on the exchange and considered how students might use the skills they have developed in their future studies and careers.

In terms of logistics, we concluded that the best way to deliver our virtual exchange was to meet all students on Zoom once a week. This allowed us to hold plenary sessions at the start of the class, before transitioning into breakout rooms where each SAGE could meet.

Different options will suit other circumstances. In high‐income countries, many universities invested money in technology to aid online and hybrid teaching during the Covid‐19 pandemic. Talk to relevant people at your institution to understand what possibilities are available. When partnering with universities in low‐resource settings, it might be necessary to work around slow internet connectivity and limited availability of technology.

## 4 Conclusions

The literature demonstrates that COIL virtual exchanges have a measurable impact on intercultural competence [1, 2, 3, 4]. We observed similar improvements in answers to questionnaires given to students at the beginning and end of the simulated pandemic exercise [13]. However, the qualitative feedback indicated that the medical students did not entirely welcome the new approach to learning. Informal discussions indicated this was related to the widely observed phenomenon that students who are comfortable in the traditional classroom tend to exhibit resistance towards new ways of learning [14]. This is rooted in fears that changes will adversely impact students by increasing their workload and making assessments harder. In the case of collaborative learning activities, students express the additional worry that their grades depend on their classmates' performance [15].

These fears are likely particularly prominent among medical students because they are less exposed to nontraditional pedagogical approaches than, say, humanities and social sciences students. In addition, medical students experience higher levels of stress than the general population [16], so they are likely to be particularly sensitive to changes in teaching and assessments. This points to the importance of clearly, explicitly and repeatedly explaining to medical students the aims and benefits of the COIL virtual exchange, with a focus on how it will benefit their future studies and careers.

## Author Contributions

**Jonathan Kennedy:** conceptualization, investigation, funding acquisition, writing – original draft. **Jessie Dubreuil:** conceptualization, investigation, funding acquisition, writing – review and editing. **Danielle Thibodeau:** conceptualization, investigation, writing – review and editing. **Aaron Zachmeier:** conceptualization, investigation, writing – review and editing.

## Conflicts of Interest

The authors declare no conflicts of interest.

## Data Availability

The data that support the findings of this study are available from the corresponding author upon reasonable request.
